# Incidence and predictors of puerperal sepsis among postpartum women at Debre Markos comprehensive specialized hospital, northwest Ethiopia: A prospective cohort study

**DOI:** 10.3389/fgwh.2023.966942

**Published:** 2023-01-24

**Authors:** Keralem Anteneh Bishaw, Yewbmirt Sharew, Endihnew Beka, Bewket Yeserah Aynalem, Liknaw Bewket Zeleke, Melaku Desta, Bekalu Kassie, Haile Amha, Tewodros Eshete, Workineh Tamir, Kerebih Bantigen, Henok Mulugeta, Addisu Andualem Ferede, Yibelu Bazezew Bitewa

**Affiliations:** ^1^ Department of Midwifery, Debre Markos University, Debre Markos, Ethiopia; ^2^ Department of Midwifery, Injibara University, Injibara, Ethiopia; ^3^ Department of Nursing, Debre Markos University, Debre Markos, Ethiopia; ^4^ Department Health Informatics, Debre Markos University, Debre Markos, Ethiopia; ^5^ Department of Laboratory, Injibara University, Injibara, Ethiopia; ^6^ School of Nursing and Midwifery, Addis Ababa University, Addis Ababa, Ethiopia

**Keywords:** puerperal sepsis, incidence, predictors, Debre Markos, Ethiopia

## Abstract

**Background:**

Puerperal sepsis is one of the leading causes of maternal mortality, particularly in low and middle-income countries where most maternal deaths occur. Women with puerperal sepsis are prone to long-term disabilities, such as chronic pelvic pain, blocked fallopian tubes, and secondary infertility. Besides this, puerperal sepsis has received less attention. For this reason, this study aimed to determine the incidence of puerperal sepsis and its predictors among postpartum women at Debre Markos Comprehensive Specialized Hospital.

**Methods:**

A prospective cohort study was conducted among 330 postpartum women from September 2020 to 2021. A pre-tested interviewer-administered questionnaire with a data extraction checklist was used to collect the data. Data were entered into Epi data 4.2 and analyzed using STATA 14.0. The incidence rate of puerperal sepsis was calculated, and a Kaplan-Meier survival curve was used to estimate the survival probability of developing puerperal sepsis. The cox-proportional hazards regression model was fitted to identify predictors of puerperal sepsis.

**Results:**

The study participants were followed for a total of 1685.3 person-week observations. The incidence rate of puerperal sepsis was 14.24 per 1,000 person-weeks. However, the overall incidence of puerperal sepsis was 7.27%. Not attending formal education [AHR: 3.55, 95% CI: (1.09–11.58)], a cesarean delivery [AHR: 4.50; 95% CI: (1.79–11.30)], premature rupture of the membranes [AHR: 3.25; 95% CI: (1.08–9.79)], complicated pregnancy [AHR: 4.80; 95% CI: (1.85–12.43)], being referred [AHR: 2.90; 95% CI: (1.10–7.65)], and not having birth preparedness and complication readiness plan [AHR: 2.95; 95% CI: (1.08–10.50)] were statistically significant predictors of puerperal sepsis.

**Conclusion:**

The incidence of puerperal sepsis was 7.27%. Not attending formal education, cesarean delivery, premature rupture of membranes, complicated pregnancy, referral status, and absence of birth preparedness and complication readiness plan were predictors associated with the incidence of puerperal sepsis.

## 1. Introduction

Puerperal sepsis is a life-threatening condition defined as organ dysfunction due to infection during pregnancy, childbirth, abortion, or after delivery (1). Postpartum infection can occur after the delivery, which has been reported as the leading cause of maternal morbidity and mortality in developing countries (2).

Between 2003 and 2009, about 73% (1,771,000) of all maternal deaths worldwide were from direct obstetric causes, and puerperal sepsis contributed to 10.7% (261,000) of all maternal deaths (3). The number of deaths from puerperal sepsis has decreased significantly in high-income countries but still accounts for the highest number of deaths in countries with limited resources. It causes at least 75,000 maternal deaths every year, mostly in low-income countries. The incidence of postpartum sepsis is relatively low in high-income countries (between 0.1 and 0.6 per 1,000 births); it is nonetheless an important direct cause of maternal mortality (10). But in developing regions such as sub-Saharan Africa and Southern Asia, major maternal deaths were due to postpartum sepsis (4).

Pre-existing maternal illnesses like (malnutrition, diabetes, obesity, severe anemia, bacterial vaginosis, and group B streptococcal infections), prolonged rupture of membranes, multiple vaginal examinations, manual placental removal, and cesarean section were significant factors associated with postpartum sepsis (4).

Prophylactic use of antibiotics for high-risk obstetric conditions such as PROM, meconium-stained amniotic fluid, perineal tears, manual placental removal, operative vaginal delivery, and cesarean section, and use of minor routine procedures (such as perineal shaving) are recommended practices to prevent morbidity and mortality caused by puerperal sepsis (4).

In Ethiopia, about 16,740 and 15,234 maternal mortality occurred in 1990 and 2013, respectively, and puerperal sepsis with other maternal infections contributed to 9.6% of maternal mortality (5). Studies in Ethiopia also reported that postpartum sepsis caused 14.68%, 21%, 26.4%, and 30.1% of maternal mortality, respectively (6–9).

In Ethiopia, maternal deaths are still high, with an estimated maternal mortality ratio of 412 maternal deaths per 100,000 live births in 2016. The causes of maternal death and individual, environmental, and health systems-related gaps contributing to maternal death in Ethiopia remain unclear (6). Moreover, very limited evidence is available in Ethiopia regarding the incidence and predictors of puerperal sepsis. Therefore, this study aimed to assess the incidence and predictors of puerperal sepsis among postpartum women at Debre Markos comprehensive specialized hospital, east Gojjam zone, Northwest Ethiopia.

## 2. Methods

### 2.1. Study area and period

The study was conducted at Debre Markos comprehensive specialized hospital (DMCSH) in Debre Markos town from September 2020 to 2021. Debre Markos town is the capital of the East Gojjam zone which is 300 km away from Addis Ababa and 265 km from Bahirdar, the capital city of Amhara National Regional State. There are 104 health centers and eight (8) district hospitals available in the catchment area of the comprehensive specialized hospital. The department of Obstetrics and Gynecology has a labour ward with six beds in the first stage room, four delivery couches in the second stage, four beds in the recovery unit, and 43 beds in the maternity ward. The ward is staffed by seven (7) obstetric and gynecological specialists, 46 midwives, one (1) emergency surgeon, one clinical midwifery specialist, 14 general practitioners, and interns.

### 2.2. Study design

An institution-based prospective follow study was conducted.

### 2.3. Source population

All postnatal women at DMCSH.

### 2.4. Study population

All postnatal women at DMCSH during the study period.

### 2.5. Inclusion and exclusion criteria

The study included women who gave birth after 28 weeks gestation. Women diagnosed with chorioamnionitis and living outside of Debre Markos town were excluded from the study.

### 2.6. Sample size determination and sampling procedure

The study sample size was estimated using Epi info version 7 with an assumption of an error rate of 5% and a power of 80% by double population proportion formula. Referral status (non-referred to referred) as an independent predictor exposure variable used since it gives the maximum sample size. The proportion among exposed group was 32.6 percent, while in the unexposed group was 16.2% (10). The overall sample size was 330 after considering a 10% non-response rate. A total of 5,400 women was delivered at DMCSH from September 2020 to September 2021. After selecting the first woman using a simple random selection (lottery) method, each research participant was selected using a systematic random sampling method.

### 2.7. Study variables

#### 2.7.1. Dependent variable

Incidence puerperal sepsis: time to event (puerperal sepsis).

#### 2.7.2. Independent variables

• **Socio-demographic factors**: Age, residence, education, monthly income, marital status.
• **Obstetric factors**: Antenatal care, place of delivery, parity, mode of delivery, premature rupture of membranes, frequency vaginal examination, prolonged labour, complication during pregnancy, birth preparedness and complication readiness plan.
• **Health facility factors**: Referral status and birth attendant.

#### 2.7.3. Operational definition

• **Puerperal sepsis**: Presence of possible, suspected, or confirmed postpartum infection plus two or more of SIRS (systemic inflammatory response syndrome) criteria; Temperature 38°C, HR > 110/M, RR > 24 B/M, WBC > 15,000 or <4,000 or >10,000 immature neutrophils, and Blood glucose >140 in absence of diabetes (10).
• **Possible, suspected, or confirmed postpartum infection**: Include either one of the following; Surgical site infections; (cesarean section site, episiotomy, genital lacerations), fever/chills, urinary tract infection, endometritis, and pelvic thrombophlebitis (10).
• **Incidence of puerperal sepsis**: The development of puerperal sepsis within the follow-up period.
• **Event**: New occurrence of puerperal sepsis at any time during the follow-up period.
• **Censored**: Women who were lost, transferred, died, or does not develop the event during the follow-up period.

### 2.8. Data collection tools and procedures

A structured checklist was designed after reviewing various literature (12, 22, and 23) to collect data from the postnatal woman, their medical card, delivery summary, and postnatal registration book. The checklist consists of baseline data such as sociodemographic characteristics, obstetrics, reproductive characteristics, vital signs, laboratory results (WBC, Hgb, and RBS), and other features. A pretest was done before the actual data collection time. The woman was evaluated for puerperal sepsis using the modified diagnostic criteria, presence of possible, suspected, or confirmed postpartum infection plus two or more SIRS criteria; Temperature 38°C, HR > 110/M, RR > 24 B/M, WBC > 15,000 or <4,000 and blood glucose >140 in the absence of diabetes (10). Participants were followed up by phone twice per week up to 6 weeks of delivery. During the follow-up period, home visits at least once a week and appointments for postnatal care per the schedule were also considered. Each postnatal woman was evaluated using a symptom questionnaire and a physical examination throughout the follow-up for puerperal sepsis. Those participants with potential puerperal sepsis findings had a WBC count and blood glucose test done at DMCSH. The data collection was carried out by trained midwives and monitored by supervisors.

### 2.9. Data quality tool

Training was given to the data collectors and supervisors before data collection. A pretest was done in Fintoselam hospital on 5% of women to modify the questionnaire 2 weeks before the actual data collection time. Regular supervision was made during data collection, and the questionnaire was reviewed and checked for completeness by the principal investigator and supervisor.

### 2.10. Statistical analysis

Data were cleaned, coded, and entered into Epi data version 4.2 and exported to STATA 14.0 for analysis. A summary statistic was applied to describe sociodemographic, obstetric, and reproductive characteristics and other variables. The incidence rate of puerperal sepsis was calculated and expressed 1,000 person-week. Kaplan-Meier survival curves were used to estimate the survival probability of developing puerperal sepsis within the follow-up period, and log-rank tests to compare the survival curves of predictor variables. Bivariable Cox proportional hazard regression was fitted, and those with a *p*-value ≤ 0.25 were fitted multivariable cox proportional hazard regression model. The necessary assumption of Cox- proportional hazard regression model fitness was checked using Schoenfeld residual and graphically by log-log plots. Adjusted hazard ratio (AHR) with 95% CI was used to assess the statistical significance and strength of the association.

## 3. Results

### 3.1. Socio-demographic characteristics of study participants

A total of 330 postnatal women were included in this prospective study. Almost fifty percent, 164 (49.7%) of the participants belonged to the (25–29) year-old age group. The median age of the study participants was 27 years with IQR (24–29) years. Almost all 325 (98.5%) participants were married. About three hundred twenty-two (97.6%) of participants were Orthodox Christian followers. One hundred fourteen 114 (34.6%) participants had a diploma and higher education. Approximately 48.2 (141) study participants had a monthly income of 1,001–3,000 ETB (Table 1).

**Table 1:** Sociodemographic characteristics of study participants at DMCSH, Debre Markos, northwest Ethiopia, 2020/2021 (*N* = 330).

Characteristics	Response	Total frequency (%)	Puerperal sepsis	
			Yes	No
Age	≤24	93 (28.2)	9 (9.7%)	84 (90.3%)
	25–29	164 (49.7)	8 (4.9%)	156 (95.1%)
	≥30	73 (22.1)	7 (9.6%)	66 (90.4%)
Marital status	Married	325 (98.5)	24 (7.4%)	301 (92.6%)
	Divorced	5 (1.5)	0 (0%)	5 (100%)
Level of education	Illiterate	40 (12.1)	7 (17.5%)	33 (82.5%)
	Primary	74 (22.4)	6 (8.1%)	68 (91.9%)
	Secondary and above	102 (30.9)	6 (5.9%)	96 (94.1%)
	Diploma and above*	114 (34.6)	5 (4.4%)	109 (95.6%)
Religion	Orthodox Christian	322 (97.6)	24 (7.5%)	298 (92.5%)
	Muslim	6 (1.8)	0 (0%)	6 (100%)
	Protestant	2 (0.6)	0 (%)	2 (100%)
Occupation	Housewife	81 (24.6)	4 (4.9%)	77 (95.1%)
	Civil servant	173 (52.4)	14 (8.1%)	159 (91.9%)
	Private business	76 (23)	10 (13.2%)	66 (86.8%)
Monthly salary	≤1,000	105 (31.8)	4 (3.8%)	101 (96.2%)
	1,001–3,000	159 (48.2)	15 (9.4%)	144 (90.6%)
	3,001–10,000	61 (18.5)	5 (8.2%)	56 (91.8%)
	>10,000	5 (1.5)	0 (0%)	5 (100%)

### 3.2. Obstetrics history, health facility, and other characteristics of study participants

More than two-thirds of the participants, 229 (69.4%), had a history of four or more antenatal care visits for recent pregnancy. Of the total study participants, 182 (55.2%) mothers were primipara. Labour started spontaneously in more than three-quarters of 273 (82.7%) postpartum women.

Approximately three-quarters, 241 (73.0%), gave birth through spontaneous vaginal delivery. More than two-thirds, 233 (70.6%) of participants had delayed labour ≥12 h. Twenty-three (7%) and thirty-four (10.3%) respondents said that their recent pregnancy was complicated by PROM and a medical/obstetric complication, respectively. Almost all 326 (98.5%) participants gave birth to a newborn baby weighing ≤4 kg. Information on the method of placental delivery and the frequency of vaginal examination is reported below in the following table (Table 2). The median baseline WBC of participants was 9.6 × 103/mm^3^ with IQR (7.8–11.2) × 103/mm^3^. The median baseline hemoglobin level of participants was 11.6 g/dl with IQR (10.2–13.2) g/dl. The study participants had a Median baseline RBS (random blood sugar) level was 102 mg/dl with IQR (90–112 mg/dl). Postpartum fever 14 (58.3%) was the most common postpartum condition observed among women with puerperal sepsis. About 7 (29.2%) women had cesarean site infection. The remaining 2 (8.3%) and 1 (4.2%) women with puerperal sepsis had episiotomy site and urinary tract infection, respectively.

**Table 2:** Obstetrics history and health facility of study participants at DMCSH, Debre Markos, northwest Ethiopia, 2020/2021 (*N* = 330).

Chacterstics	Response	Total frequency (%)	Puerperal sepsis	
			Yes *N* (%)	No *N* (%)
Frequency of ANC visit	≤3	101 (30.6)	16 (15.8%)	85 (84.2%)
	≥4	229 (69.4)	8 (3.5%)	221 (96.5%)
Parity	Primipara	182 (55.2)	13 (7.1%)	169 (92.9%)
	Multipara	148 (44.8)	11 (7.4%)	137 (92.6%)
Onset of labour	Spontaneous	273 (82.7)	19 (7%)	254 (93%)
	Induced	28 (8.5)	3 (10.7%)	25 (89.3%)
	Not started	29 (8.8)	2 (6.9%)	27 (93.1%)
Mode of delivery	Spontaneous vaginal delivery	241 (73.0)	9 (3.7%)	232 (96.3%)
	Instrumental	14 (4.3)	2 (14.3%)	12 (85.7%)
	Caesarean section	75 (22.7)	13 (17.3%)	62 (82.7%)
Duration of labour	<12 h	233 (70.6)	17 (7.3%)	216 (92.7%)
	≥12 h	97 (29.4)	7 (7.2%)	90 (92.8%)
PROM	Yes	23 (7%)	5 (21.7%)	18 (78.3%)
	No	307 (93%)	19 (6.2%)	288 (93.8%)
Method of placenta delivery	CCT	265 (80.3)	16 (6%)	249 (94%)
	Manual	65 (19.7)	8 (12.3%)	57 (87.7%)
Birth weight	≤4 kg	326 (98.5)	22 (6.8%)	304 (93.2%)
	>4kg	4 (1.5)	2 (50%)	2 (50%)
Frequency of vaginal examination	≤4	221 (67)	13 (5.9%)	208 (94.1%)
	≥5	109 (33)	11 (10.1%)	98 (89.9%)
Complication during pregnancy	Yes	34 (10.30)	7 (20.6%)	27 (79.4%)
	No	296 (89.7)	17 (5.7%)	279 (94.3%)
Types of medical health problem (*N* = 34)	Anemia	10 (18.5%)	2 (20%)	8 (80%)
	Preeclampsia	8 (18.5%)	2 (25%)	6 (75%)
	Diabetes miltus	3 (11.1%)	2 (66.7%)	1 (33.3%)
	Twin pregnancy	6 (22.2%)	1 (16.7%)	5 (83.3%)
	Asthma	1 (3.7%)	0 (0%)	1 (100%)
	Hepatitis	1 (3.7%)	0 (%)	1 (100%)
	Syphilis	1 (3.7%)	0 (%)	1 (100%)
	HIV/AIDS	4 (14.8%)	0 (%)	4 (100%)
Birth attendant	Health professional	327 (99)	24 (7.3%)	303 (92.7%)
	TBA	3 (1)	0 (0%)	3 (100%)
Referral status	Yes	58 (17.6)	8 (13.8%)	50 (86.2%)
	No	272 (82.4)	16 (5.9%)	256 (94.1%)
BPCR	Yes	129 (39.1)	4 (3.1%)	125 (96.9%)
	No	201 (60.1)	20 (10%)	181 (90%)

Almost all 327 (99%) participants were assisted by skilled birth attendants during delivery. More than one-third, of 129 (39.1%) postnatal women reported developing BPCR. Regarding referral status, Fifty-eight (17.6) participants were referred to DMCSH after labour started (Table 2).

### 3.3. Incidence of puerperal sepsis

The study participants followed for a minimum and maximum of 0.143 weeks and 6 weeks, respectively. During the follow-up period, 24 (7.27%) new cases of puerperal sepsis were noted, and the remaining 306 (92.73%) were censored. The median follow-up time was 6 weeks with an interquartile range of (IQR: 4.6–6 weeks). The incidence rate of puerperal sepsis was 14.24 per 1,000 person-weeks (PWs) (95% CI = 9.55–21.25) with a total of 1685.3 patient-week observations. Among 24 postnatal women reporting puerperal sepsis, 7 (29.2%) were complicated pregnancies. Of these, 2 (28.6%) had anemia, preeclampsia, and diabetes Miletus.

A higher incidence, 16 (66.7%) and 13 (54.2%) of puerperal sepsis cases, were reported in postnatal women who had ≤3 ANC visits and cesarean delivery, respectively. In addition, the incidence of puerperal sepsis was 11 (45.8%) and 5 (20.8%) among women who had PROM and vaginal examinations of ≥5, respectively. Moreover, one-third (8) incidence of puerperal sepsis occurred among referred postnatal women.

### 3.4. Kaplan-Meier survival analysis

**Figure 1:**
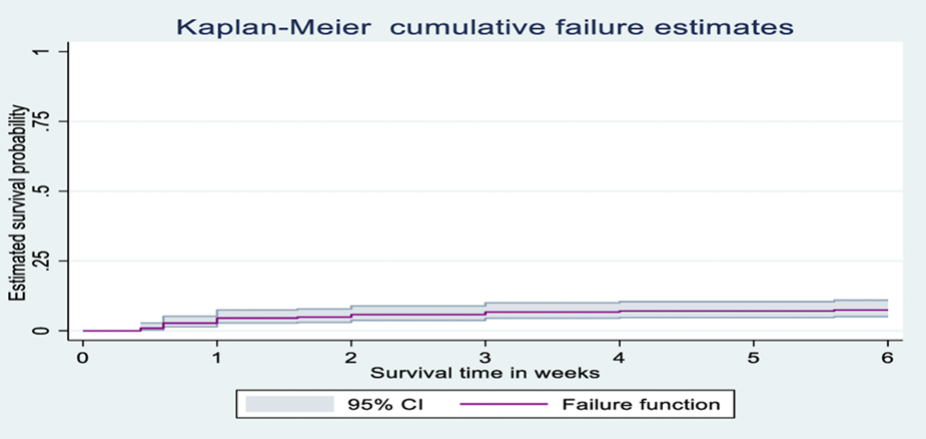
Kaplan-Meier curve showing the survival probability of developing puerperal sepsis.

**Figure 2:**
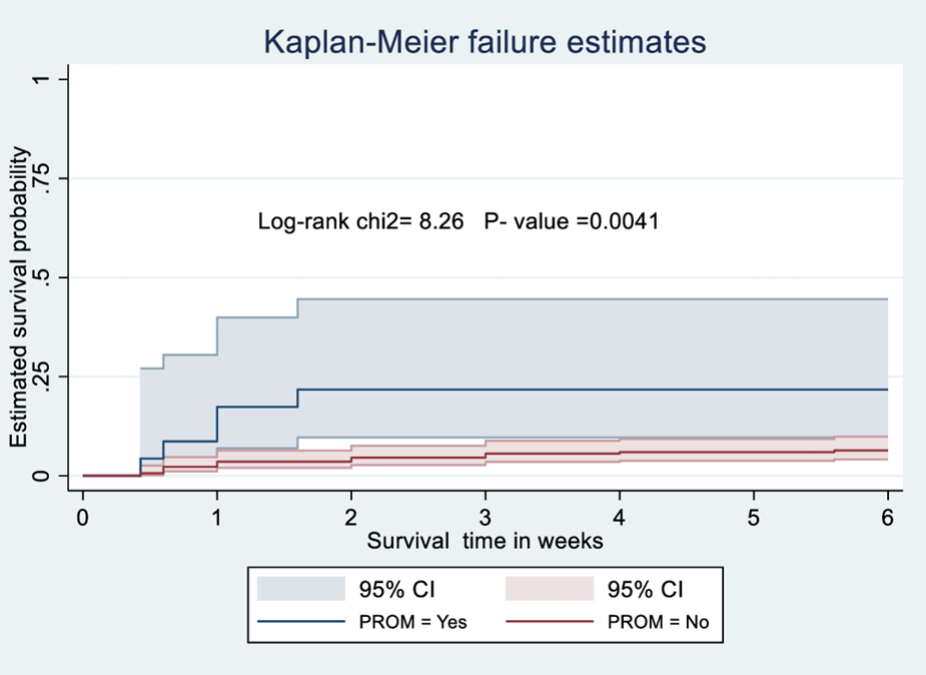
Kaplan-Meier curve showing the survival probability of developing puerperal sepsis among postnatal women based on PROM.

**Figure 3:**
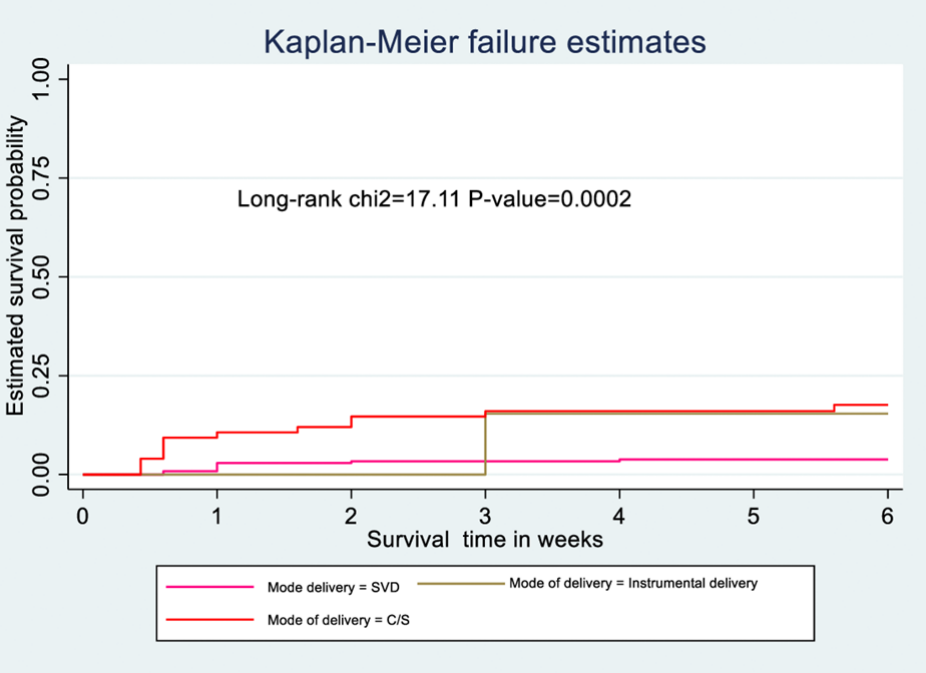
Kaplan-Meier curve showing the survival probability of developing puerperal sepsis among postnatal women based on the mode of delivery.

**Figure 4:**
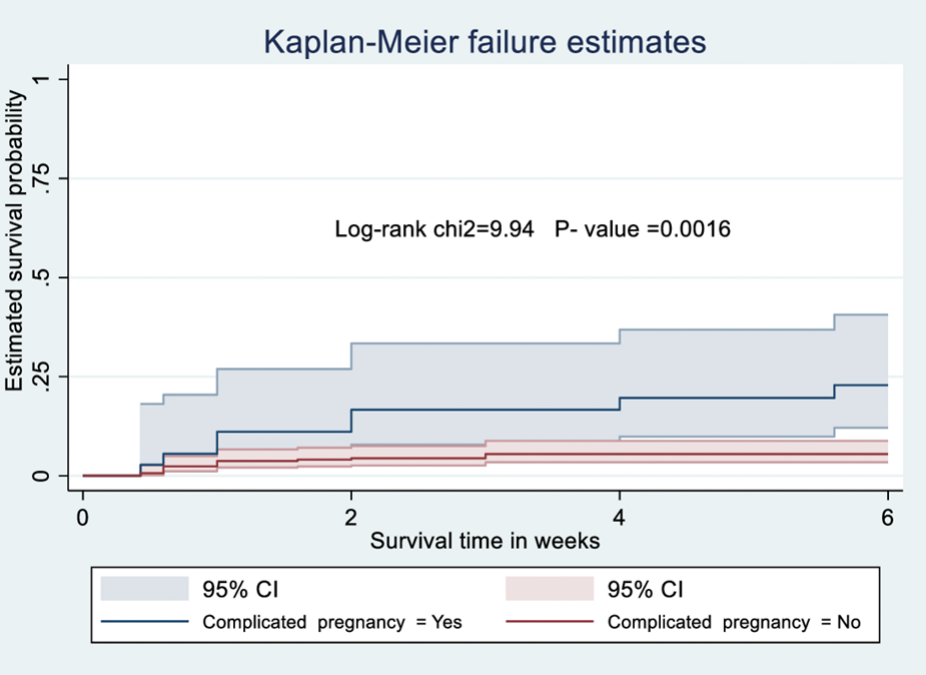
Kaplan-Meier curve showing the survival probability of developing puerperal sepsis among postnatal women based on pregnancy condition.

**Figure 5:**
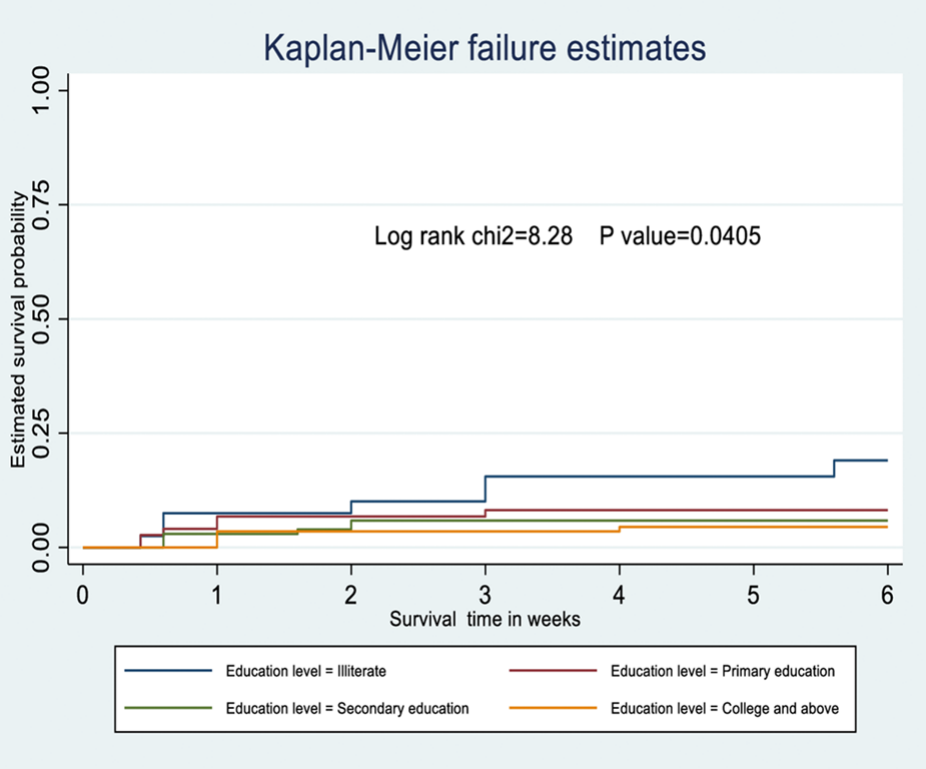
Kaplan-Meier curve showing the survival probability of developing puerperal sepsis among postnatal women based on the education level.

**Figure 6:**
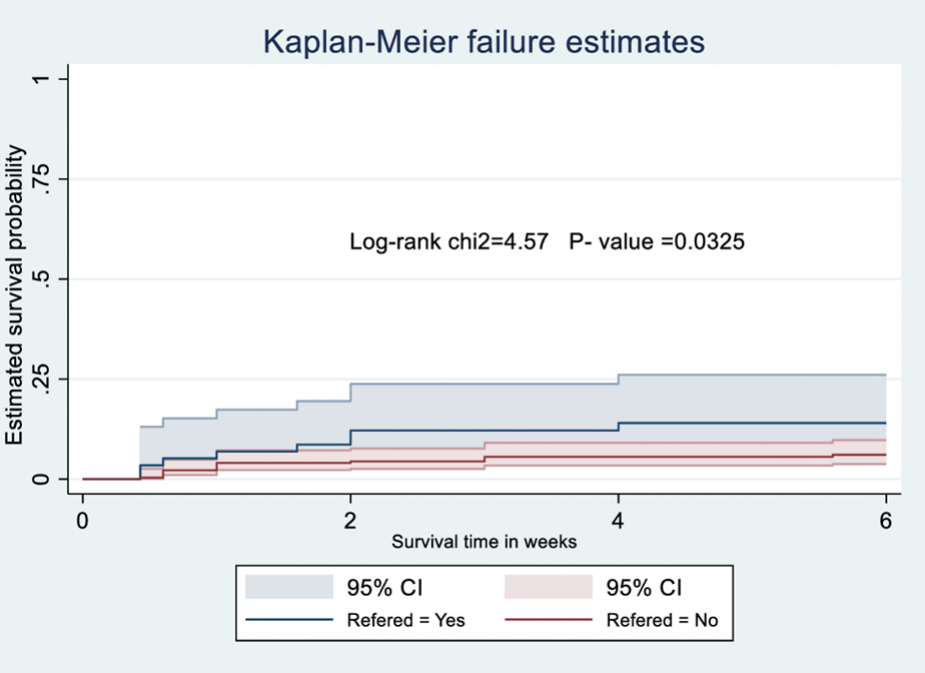
Kaplan-Meier curve showing the survival probability of developing puerperal sepsis among postnatal women based on referral status.

**Figure 7:**
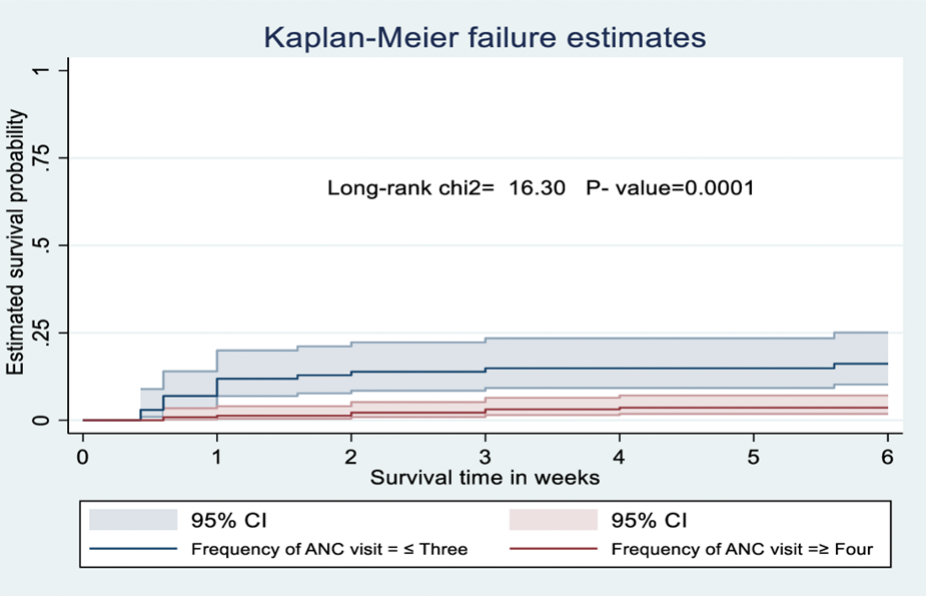
Kaplan-Meier curve showing the survival probability of developing puerperal sepsis among postnatal women based on the frequency of ANC visit.

**Figure 8:**
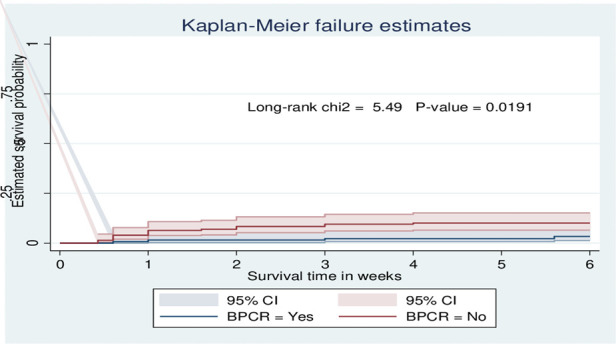
Kaplan-Meier curve showing the survival probability of developing puerperal sepsis among postnatal women based on BPCR.

The probability of surviving from delivery to puerperal sepsis within 6-week follow-up period was estimated. The Kaplan-Meier survival curve showed that the probability of developing puerperal sepsis decreased over time. Most cases of puerperal sepsis occurred within (three) 3 weeks of the follow-up period (Figure 1). A higher probability of developing puerperal sepsis developed among mothers with PROM (Figure 2), delivered by c/s (Figure 3), and complicated pregnancy (Figure 4). A relatively higher probability of developing puerperal sepsis occurred among mothers without formal education (Figure 5). A relatively lower rate of developing puerperal sepsis was reported among mothers who referred (Figure 6), ANC visit ≤3 (Figure 7). The lowest probability of developing puerperal sepsis occurred among mothers who did not develop a BPCR plan (Figure 8).

### 3.5. Predictors of puerperal sepsis

Educational level, frequency of ANC visit, mode of delivery, PROM, method of placental delivery, complicated pregnancy, BPCR plan, and referral status were predictors of puerperal sepsis at *p*-value ≤0.25 in bivariate Cox regression. But only educational level, frequency of ANC visits, mode of delivery, PROM, complicated pregnancy, BPCR plan, and referral status remained statistically significant with puerperal sepsis in the multivariable cox regression model. The cox-proportional hazard assumptions for these variables was a (global test, *p*-value = 0.403).

This study revealed that women who did not attend formal education were 3.55 times higher risk of developing puerperal sepsis as compared to women who had a diploma and above education level (adjusted hazard ratio (AHR) = 3.55: 95% confidence interval (CI): 1.09–11.58). Women with one to three ANC visits were 4.52 times higher risk of developing puerperal sepsis compared to women with four and above times of ANC visits [AHR: 4.52; 95% CI: (1.86–10.98)]. Similarly, women who delivered by cesarean section (C/S) were 4.5 times higher risk of developing puerperal sepsis compared to delivered by spontaneous vaginal delivery [AHR: 4.50; 95% CI: (1.79–11.30)].

Regarding premature rupture of amniotic membrane (PROM), having PROM before delivery had 3.25 times more risk to develop puerperal sepsis in postnatal women as compared to the counterpart [AHR: 3.25; 95% CI: (1.08–9.79)]. Complicated pregnancy was another factor associated with an increased risk of puerperal sepsis. Those women with complicated pregnancies were 4.80 times more at risk of developing puerperal sepsis than those who had uncomplicated pregnancies [AHR: 4.80; 95% CI: (1.85–12.43)].

Finally, the risk of developing puerperal sepsis among women who were referred from other institutions and did not have BPCR were 2.90 [AHR: 2.90; 95% CI: (1.10–7.65)] and 2.95 [AHR: 2.95; 95% CI: (1.08–10.50)] times as compared to the counterpart respectively (Table 3).

**Table 3:** Cox regression analysis predictors of puerperal sepsis among postnatal women at DMCSH, Debre Markos, northwest Ethiopia, 2020/21 (*N* = 330).

Variables	Response	Survival status		CHR (*p*-value)	AHR (95% CI)
		Event	Censored		
Level of education	Not attending formal education	7 (17.5%)	33 (82.5%)	4.21 (0.014)	3.55 (1.09–11.58)
	Primary	6 (8.1%)	68 (91.9%)	1.87 (0.300)	1.18 (0.33–4.24)
	Secondary	6 (5.9%)	96 (94.1%)	1.35 (0.624)	0.78 (0.22–2.74)
	Diploma and above*	5 (4.4%)	109 (95.6%)	1	1
Frequency of ANC visit	≤3	16 (15.8%)	85 (84.2%)	4.78 (0.001)	4.52 (1.86–10.98)
	≥4	8 (3.5%)	221 (96.5%)	1	1
Mode of delivery	Spontaneous vertex delivery	9 (3.7%)	232 (96.3%)	1	1
	Instrumental	2 (14.3%)	12 (85.7%)	3.78 (0.089)	2.34 (0.47-11.77)
	Caesarean section	13 (17.3%)	62 (82.7%)	4.87 (0.001)	4.50 (1.79-11.30)
PROM	Yes	5 (21.7%)	18 (78.3%)	3.76 (0.008)	3.25 (1.08–9.79)
	No	19 (6.2%)	288 (93.8%)	1	1
Method of placenta delivery	CCT	16 (6%)	249 (94%)	1	1
	Manual	8 (12.3%)	57 (87.7%)	2.13 (0.081)	0.94 (0.36-2.43)
Complication during pregnancy	Yes	7 (20.6%)	27 (79.4%)	3.93 (0.002)	4.8 (1.85–12.43)
	No	17 (5.7%)	279 (94.3%)	1	1
BPCR	Yes	8 (13.8%)	50 (86.2%)	1	1
	No	16 (5.9%)	256 (94.1%)	3.29 (0.030)	2.95 (1.08-10.50)
Referral status	Yes	4 (3.1%)	125 (96.9%)	2.45 (0.039)	2.90 (1.10-7.65)
	No	20 (10%)	181 (90%)	1	1

## 4. Discussion

This is the first prospective follow-up study conducted in Ethiopia to determine the incidence and predictors of puerperal sepsis among postnatal women at DMCSH. The incidence rate of puerperal sepsis was 14.24 per 1,000 person - weeks. The present study reported that the overall incidence of puerperal sepsis was 7.27% (95% CI: 4.47–10.01). This finding is in line with studies conducted in the USA (6%) (11) and India (8.68%) (12).

The finding of this study is significantly higher as compared to studies in Pakistan (1.7% and 3.89%) (13, 14), Vietnam (1.7%) (15), India (3.9%) (16), Ireland (0.18%) (17), USA (0.0294) (18), Nigeria (0.78%, and 0.9%) (2, 19) Uganda (2%) (20). The reasons for this discrepancy might be due to variations in the socio-demographic characteristics, study period, and study population. Disparities in health service utilization and accessibility and variations in the quality of maternal health services during the intrapartum period may contribute to this difference. The better quality of antenatal and intrapartum care in developed countries, resulting in a lower incidence of puerperal sepsis, may also contribute to this difference. The high incidence of puerperal sepsis in this setting can be attributed to the higher number of cases in the hospital as it is the only comprehensive specialized hospital in the East Gojjam zone. Being a referral center for complicated and high-risk pregnancies may also contribute to a higher incidence of puerperal sepsis in this hospital due to complicated and high-risk pregnancies are at risk of developing puerperal sepsis. The methodological difference may also contribute to this discrepancy.

On the other hand, it is lower when compared with a study in Kenya (12.2%) (21). The difference in the study setup and time difference between the studies may contribute to the discrepancy.

The hazard of developing puerperal sepsis was 3.55 among mothers who didn't attend formal education compared to those with a diploma and above education level. This result is consistent with studies in Ethiopia (22), Pakistan (13), and California (23), as postnatal women with a lower level of education level were at a significantly greater risk of developing puerperal sepsis. This might be due to educated women having healthier reproductive practices, good health-seeking behavior, and may avoid delays in seeking medical care to minimize the risk of complicated pregnancy. In addition, educated women may have a positive attitude about health care, nutrition, and healthier behaviors.

This finding has significant clinical implications for reducing puerperal sepsis incidence and its impact by preventing puerperal sepsis through avoiding delay in seeking maternal health care services due to lower educational level and poor knowledge. Given that a low educational level was associated with inadequate knowledge and self-care practices regarding puerperal sepsis, this finding suggested that postnatal mothers who did not attend formal education should receive special attention. This is because good knowledge and self-care practices strongly recommend for preventing puerperal sepsis (24). From a policy perspective, ensuring obstetric caregivers have the knowledge and skills to provide intensive training regarding puerperal sepsis, especially to postnatal women with a low level of education. Improving girls’ enrollment in and completion of school through empowering females and the community using gender-inclusive and responsive programs to reduce such type's low-level educational accomplishment should be considered. Integrating intensive health education programs for women of reproductive age during the preconception period and ANC is one method to improve their awareness of issues like puerperal sepsis and other maternal health problems.

The study revealed that mothers with one to three ANC visits were 4.52 times more likely to develop puerperal sepsis than those with ≥4 ANC visits. Studies in Uganda (20) and Nigeria supported this finding (19). This could be due to the importance of ANC in preventing puerperal sepsis by detecting and treating disease conditions/risk factors earlier that result in postpartum infection, such as urinary tract infection, anemia, sexually transmitted infection, and PROM. The importance of ANC in health promotion and disease prevention through tetanus immunization, iron and folate supplementation, malaria prevention with ITN, good nutrition, protection against iodine deficiency, risks of using tobacco, alcohol, and traditional remedies, hygiene, and infection prevention practices may also contribute to a lower incidence of puerperal sepsis.

The study revealed that mothers who gave birth by C/S were 4.5 times higher risk of developing puerperal sepsis than those delivered by SVD. This finding is similar to studies in Kenya (21), Nigeria (19), Uganda (20), Tanzania (25), Nigeria (26), India (12), Germany (27), California (23), and Ireland (17). This is because early puerperal infectious morbidity is common after a cesarean birth (28). And also, it could be due to multiple vaginal examinations, prolonged labour and prolonged duration of operation, prolonged time of ruptured membranes, excessive blood loss, the poor intervention of preventive measures, poor prenatal care, and the presence of other co-morbidities, like obesity, diabetes, HIV, chorioamnionitis, and previous cesarean deliveries.

Because postnatal education is critical in assisting women to recognize the early warning signs of puerperal sepsis, obstetric caregivers should educate postnatal women about early warning signs and potential complications of cesarean birth, including puerperal sepsis, as part of hospital discharge. This result has important clinical implications for reducing the risk of cesarean site infection using evidence-based safety measures such as vaginal cleaning, skin preparation, timely antibiotic administration, and maintenance of thermoregulation. This finding is vital for policymakers to adopt the enhanced recovery after cesarean protocol and to incorporate it into routine care for women having a cesarean birth.

In this study, PROM during pregnancy had 3.25 times the risk of developing puerperal sepsis in postnatal women compared to the counterpart. This evidence is consistent with studies in India (12) and Pakistan (14). The possible reason may be the impairment of natural mechanical barriers, such as a ruptured amniotic sac and prolonged opening of the cervix, creating a favorable condition for microorganisms to ascend and cause infection.

Complicated pregnancy was another factor associated with an increased risk of puerperal sepsis. Women with complicated pregnancies were 4.80 times more likely to develop puerperal sepsis compared to the counterpart. Studies in Egypt (29) and the USA (18) support this evidence, as anemia and Eclampsia were associated with maternal sepsis.

This study also revealed that the risk of developing puerperal sepsis among mothers referred was 2.95 times compared to the counterpart. This result is consistent with a study in Ethiopia (22). The finding has important clinical implications for enhancing referral conditions by increasing access to communication and transportation systems to make referral effective and efficient and to improve the quality of care by ensuring the readiness of high-level healthcare facilities (referral site). The finding is also significant for policymakers to consider protocols that meet the need of families who do not comply with referral advice to avert the need for referral by upgrading initial care provided by obstetric caregivers at low-level health facilities (30).

Lastly, according to this study, women who did not have a BPCR plan were 2.90 times more likely to develop puerperal sepsis than women who had a BPCR plan. This could be due to the importance of BPCR to avoid delaying women's health-seeking behavior by anticipating and recognizing obstetric emergencies and complications at an early stage. BPCR is a fundamental intervention to improve maternal health in developing countries (31).

## 5. Limitations of the study

First and foremost, this is the first study conducted to investigate the incidence of and predictors of puerperal sepsis, with a follow-up study at DMCSH. Because the study was conducted at the zone's only tertiary level of care, the true incidence of puerperal sepsis in the region may elevate. The results may not accurately reflect the figure of primary and general hospitals. Missed cases may occur because postnatal women are not always accessible throughout the follow-up period.

## 6. Conclusion and recommendations

The incidence of puerperal sepsis was 7.27%. Not attending formal education, lower frequency of ANC visits, c/s delivery, PROM, complicated pregnancy, not having a BPCR plan, and being referred were associated with the incidence of puerperal sepsis. Therefore, a collaborative effort is needed at the community and health institutions level to minimize complications during pregnancy and delivery and enable pregnant women to develop a birth preparedness and complication readiness plan. Interventions to avoid PORM and complicated pregnancy are required. And also, Responsible bodies should implement strategies to improve the number of ANC visits, referral conditions, and educational status of women.

Overall, this study has the potential to be a very useful reference for planners of midwifery care and education in low-income countries to reduce the occurrence of puerperal sepsis and its contribution to maternal morbidity and mortality.

## Data Availability

The raw data supporting the conclusions of this article will be made available by the authors, without undue reservation.
